# Supplementary figures and images for: Thermal Cycling of (RE)BCO-Based Superconducting Tapes Joined by Lead-Free Solders
Source: Materials (Basel). 2021 Feb 23;14(4):1052. doi: 10.3390/ma14041052 (PMC7926784; doi:10.3390/ma14041052)

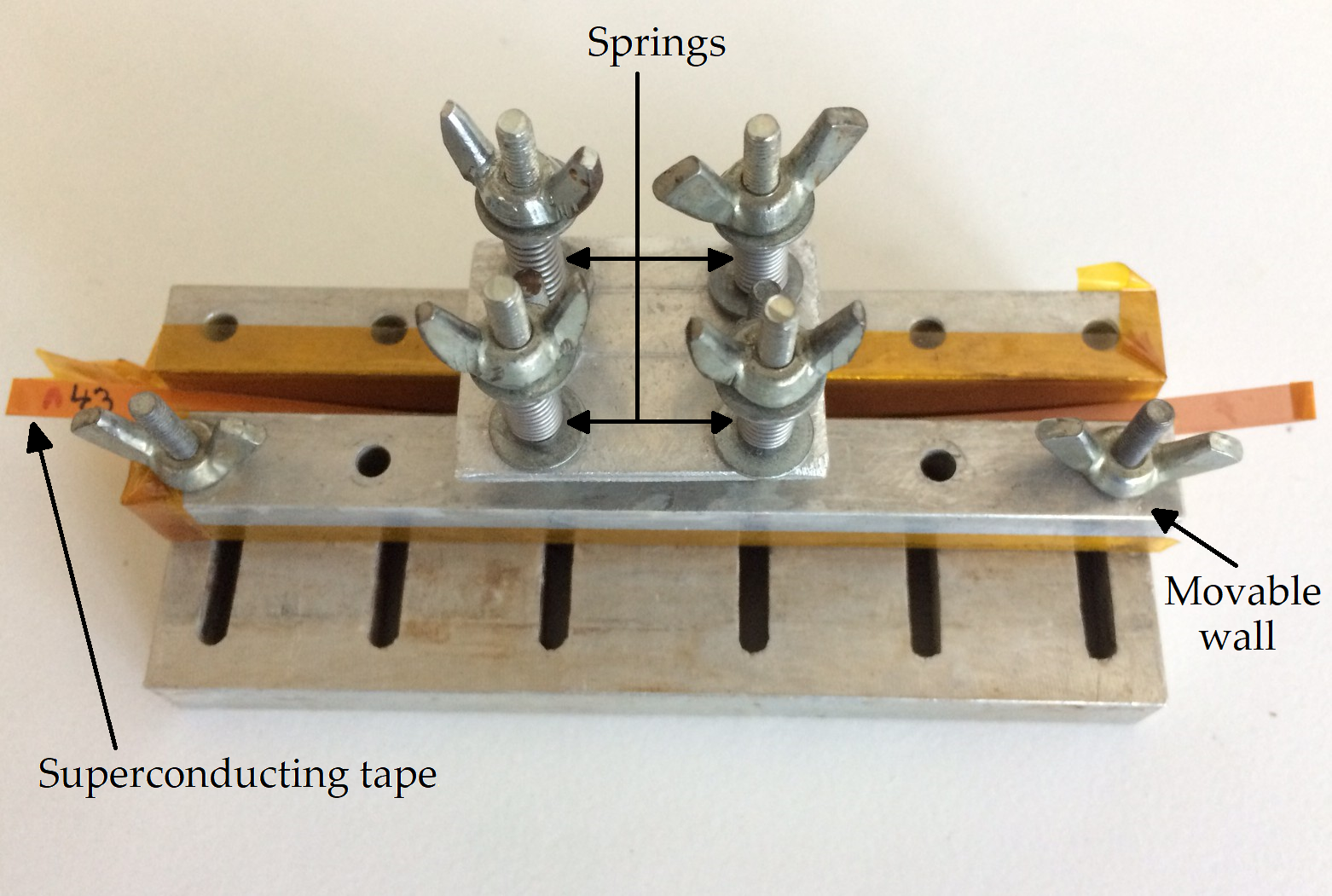

Supplement: Supplementary file 1 [file materials-14-01052-s001.zip › FigureS01.tif]

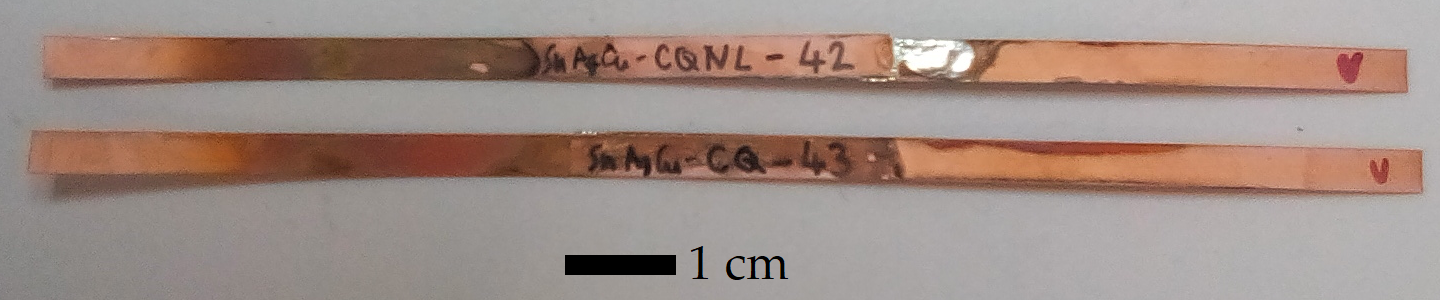

Supplement: Supplementary file 1 [file materials-14-01052-s001.zip › FigureS02.tif]

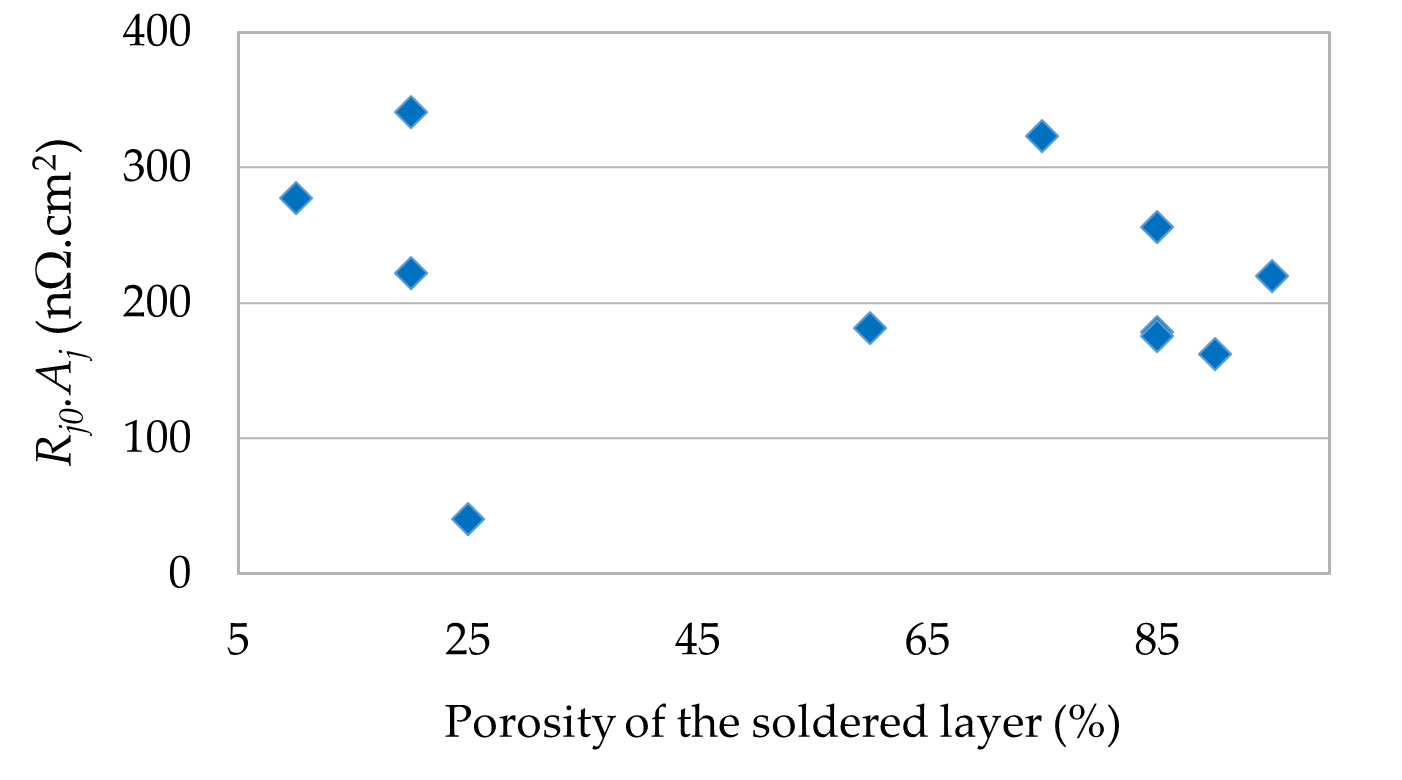

Supplement: Supplementary file 1 [file materials-14-01052-s001.zip › FigureS03.tif]

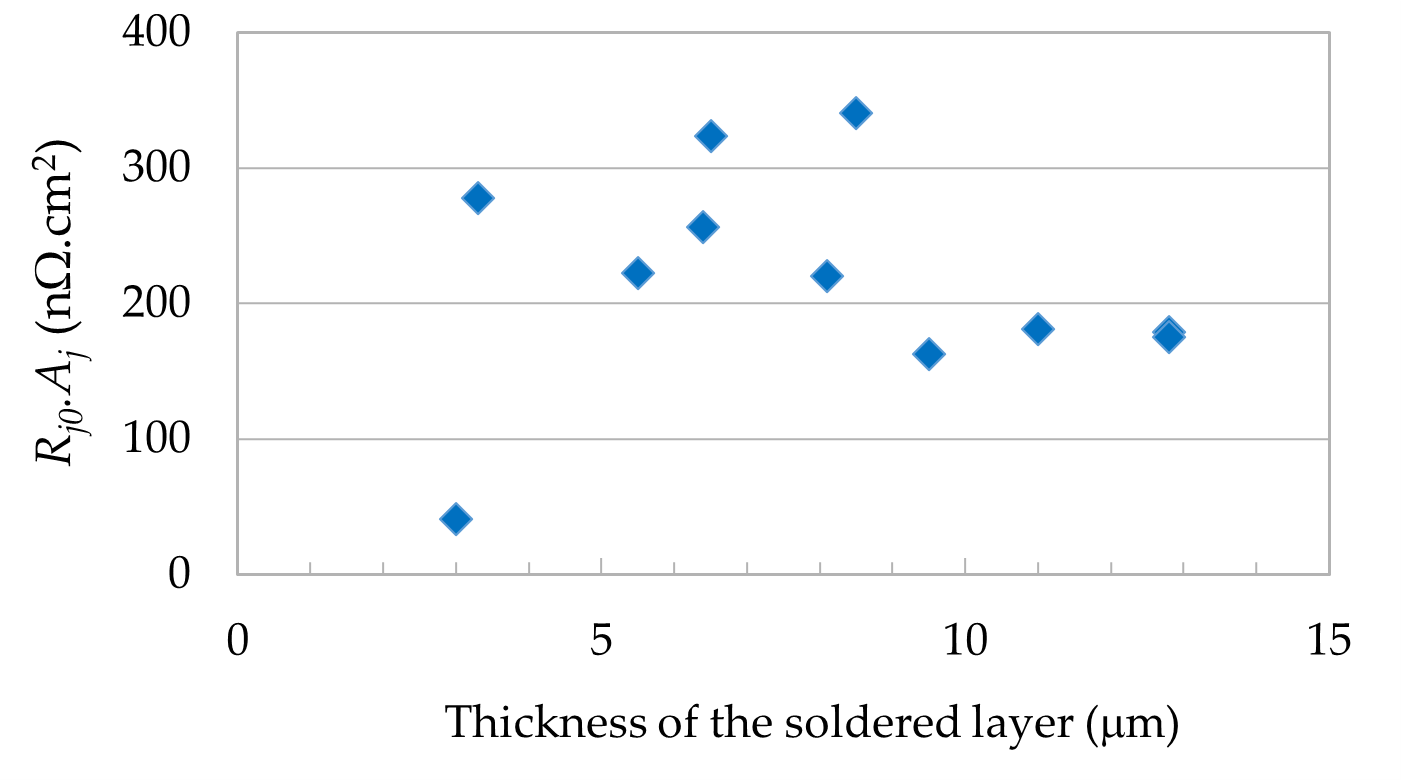

Supplement: Supplementary file 1 [file materials-14-01052-s001.zip › FigureS04.tif]

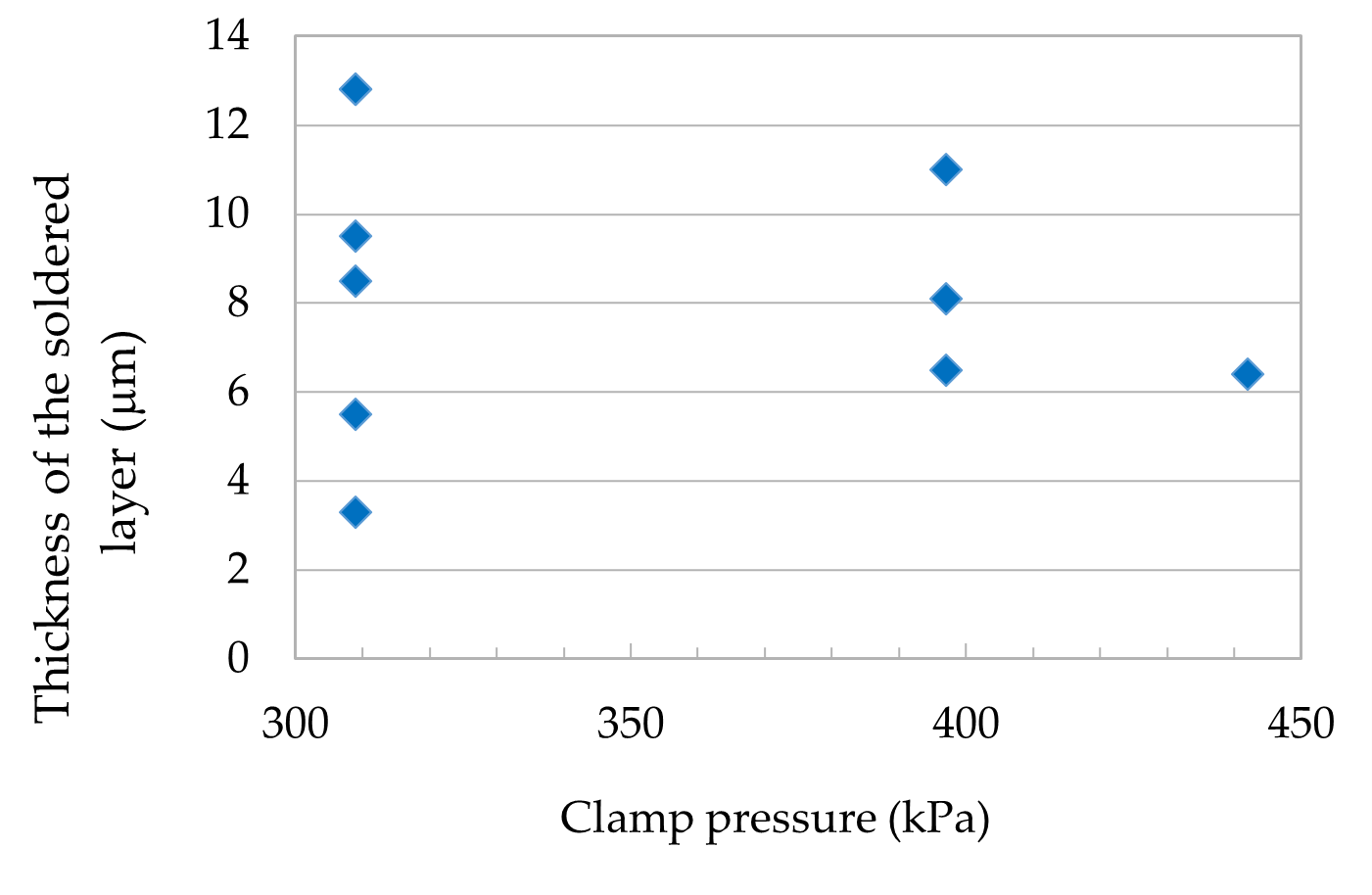

Supplement: Supplementary file 1 [file materials-14-01052-s001.zip › FigureS05.tif]

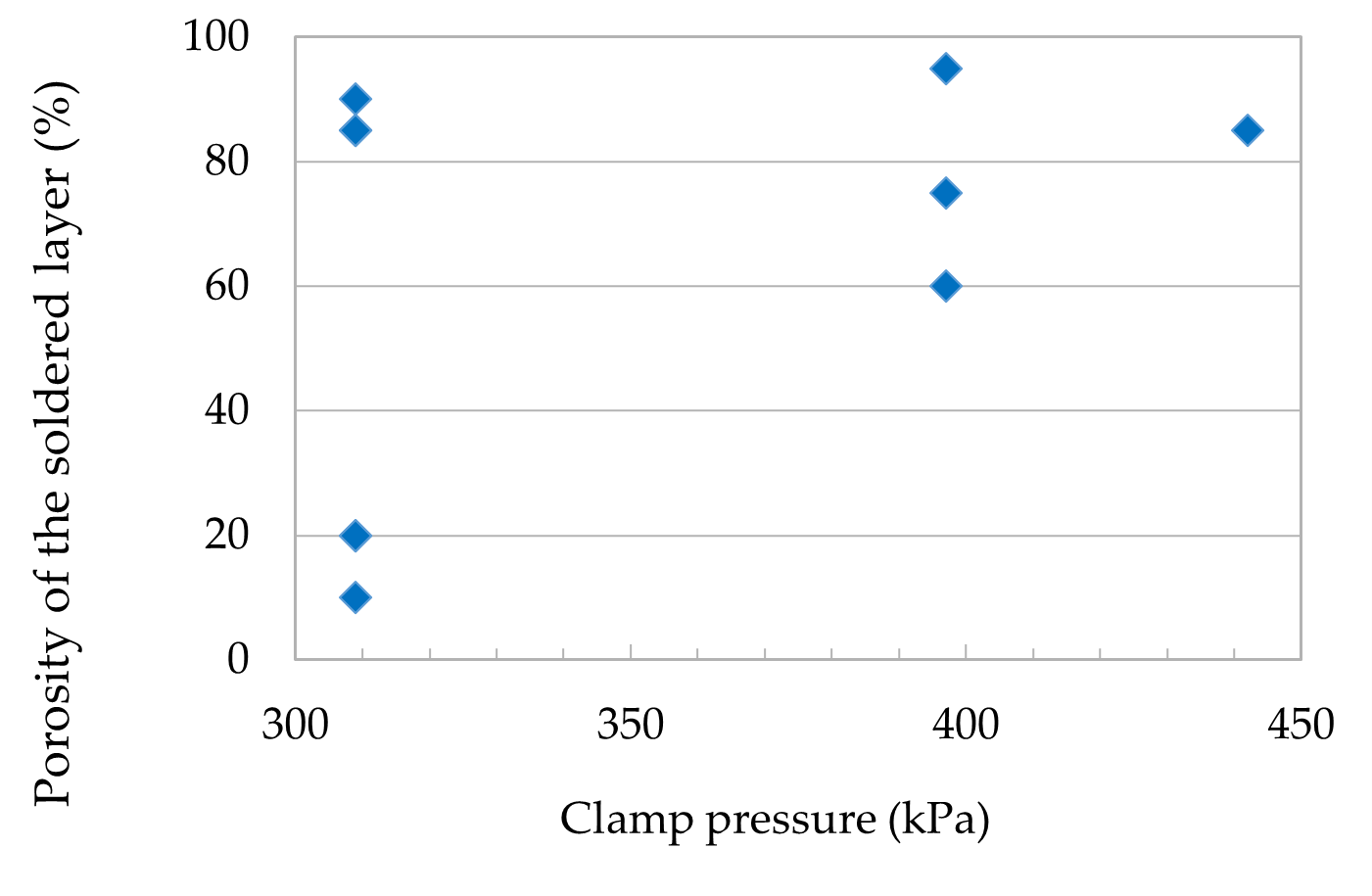

Supplement: Supplementary file 1 [file materials-14-01052-s001.zip › FigureS06.tif]

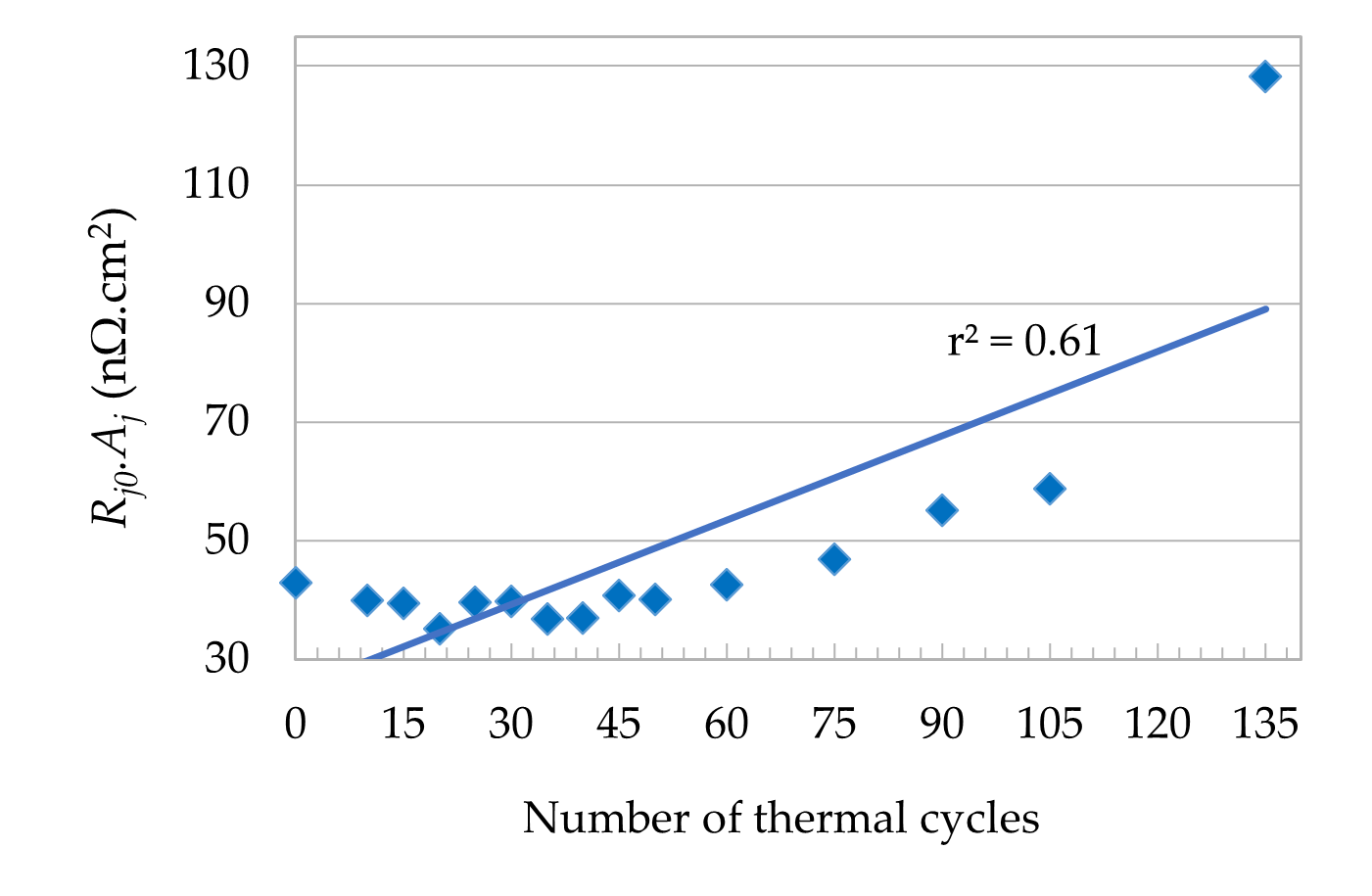

Supplement: Supplementary file 1 [file materials-14-01052-s001.zip › FigureS07.tif]

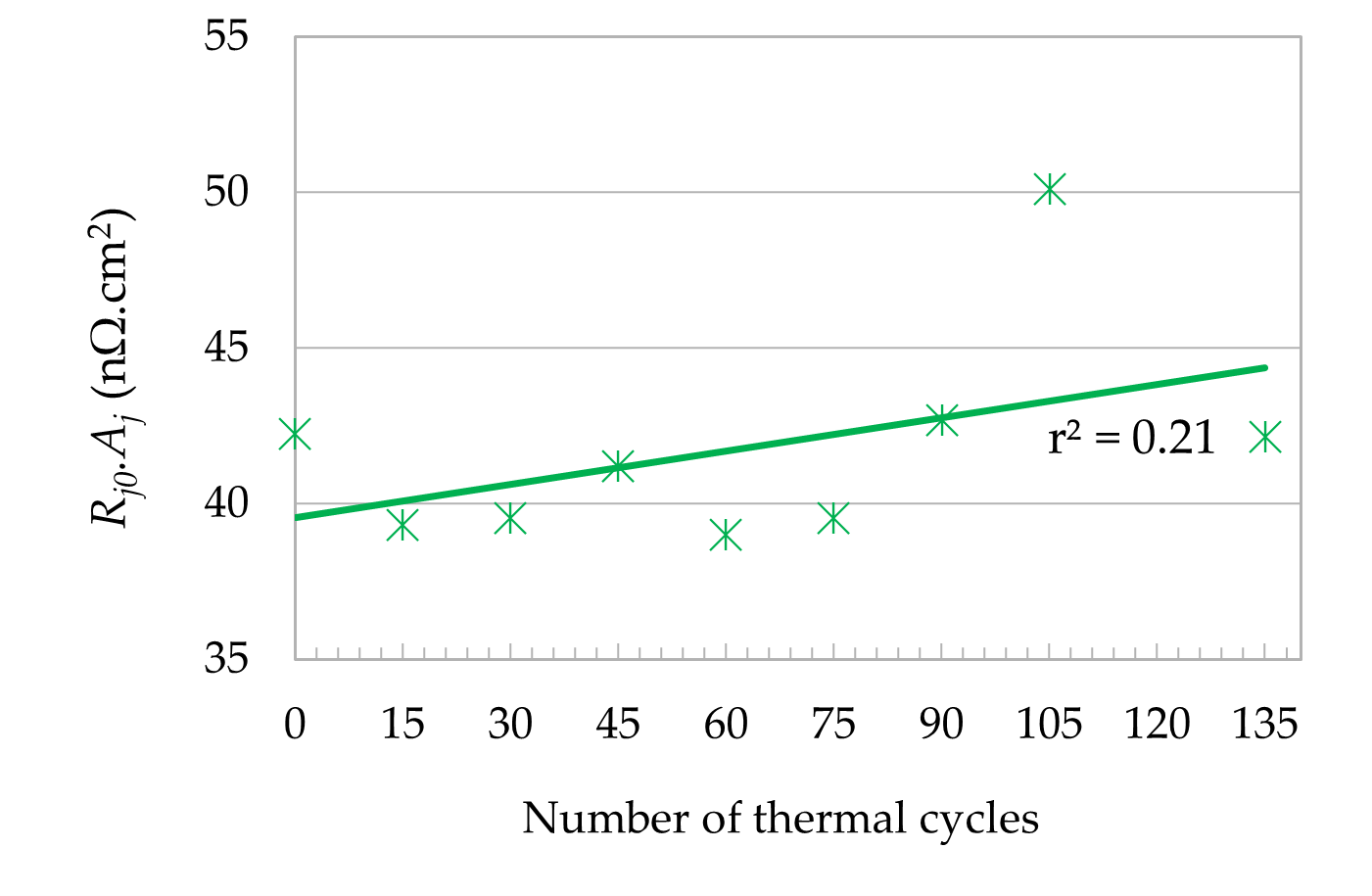

Supplement: Supplementary file 1 [file materials-14-01052-s001.zip › FigureS08.tif]

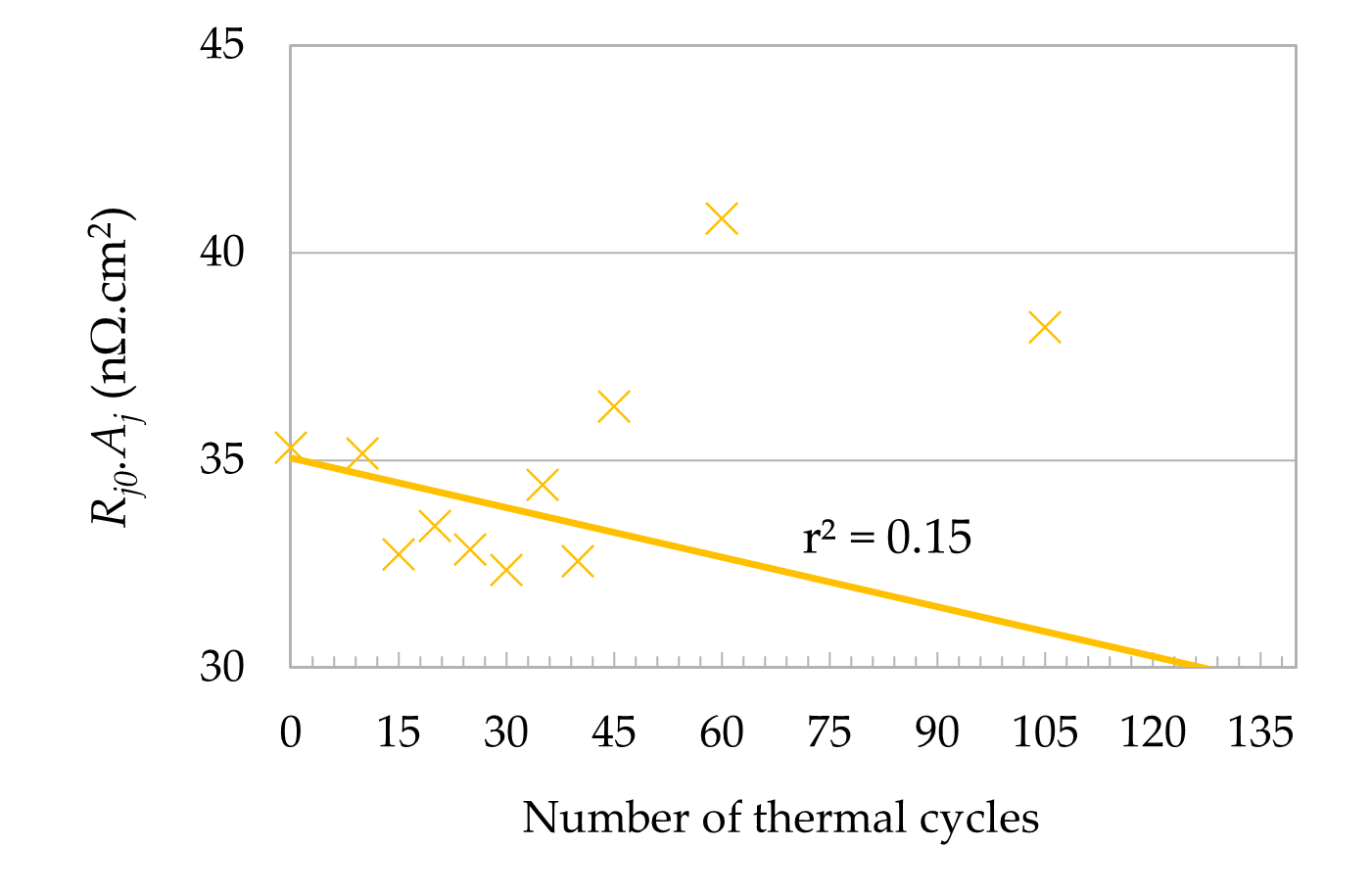

Supplement: Supplementary file 1 [file materials-14-01052-s001.zip › FigureS09.tif]

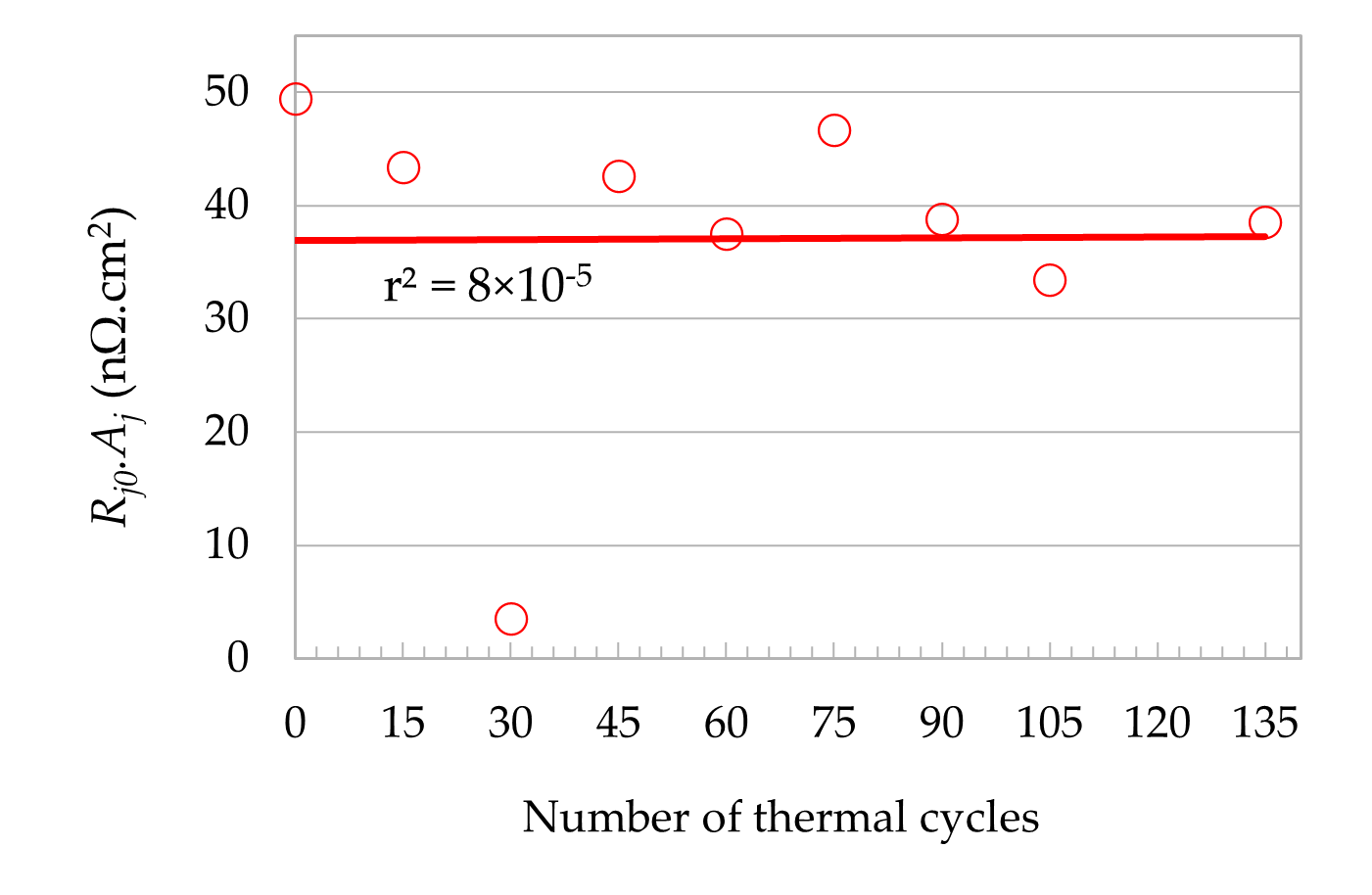

Supplement: Supplementary file 1 [file materials-14-01052-s001.zip › FigureS10.tif]
